# How to draw the line between partial and radical nephrectomy

**DOI:** 10.1590/S1677-5538.IBJU.2020.0149.1

**Published:** 2021-07-20

**Authors:** Diego Moreira Capibaribe, Marcos Oliveira Siebra Coelho, Leonardo O. Reis

**Affiliations:** 1 Pontifícia Universidade Católica de Campinas Divisão de Oncologia Urológica CampinasSP Brasil Divisão de Oncologia Urológica, Pontifícia Universidade Católica de Campinas - PUC-Campinas, Campinas, SP, Brasil; 2 Pontifícia Universidade Católica de Campinas Laboratório de UroSciências CampinasSP Brasil Laboratório de UroSciências, Pontifícia Universidade Católica de Campinas - PUC-Campinas, Campinas, SP, Brasil; 3 Universidade Estadual de Campinas Departamento de Urologia CampinasSP Brasil Departamento de Urologia, Universidade Estadual de Campinas - Unicamp, Campinas, SP, Brasil

## COMMENT

There has been discussion over where to draw the line between partial and radical nephrectomy ever since performing the surgery was deemed possible. In 1869 Gustav Simon made history by performing the first ever planned nephrectomy to cure a urinary fistula and later in 1870, the first partial nephrectomy to treat hydronephrosis (1). That stated two important facts that are pertinent to our discussion. The first statement was that removing a kidney or part of one was possible. The second statement affirmed that it was possible to live with only one functioning organ. With that in mind, we persist year after year, trying to figure out where to put a line.

Beyond oncological control, the risk of chronic kidney disease, cardiovascular events, hospitalization and death are problems that we as urologists must keep in mind when discussing long term repercussions of kidney cancer treatment, to find a way to push it as far as possible from our patients waiting for a partial or radical nephrectomy (2).

Many studies have shown (3-6) that locally invasive tumors such as T3a can be resected in a nephron sparing surgery (NSS) with oncological safety in long enough follow-up. Although positive surgical margins do increase with NSS of more complex and advanced tumors, their consequences are still negligible and a two year follow up, although reduced, is probably enough time to evaluate properly a recurrence rate based on previous studies (7).

Still, the literature is teeming with retrospective, non-randomized, biased filled works that try to give us some direction but are yet to give us any definitive answer. With that in mind, one other aspect to discuss would be the benefit of NSS and renal function preservation in this scenario. The follow up becomes central when dealing with this subject, once it has been reported that average time to recover original kidney function rate could take up to 25 months for 49% of patients to regain their previous eGFR (8, 9).

Tumor size is also significantly different between most partial and radical nephrectomy studied groups, and that may also impact in the final renal function recovery (10). Many studies have shown even in the same T stage, that size may interfere in terms of benefit when performing NSS. According to de Andrade et al. (11) who analyzed patients submitted to radical nephrectomy, it was found that patients with kidneys with larger tumors suffered lower eGFR decreases when compared to kidneys with smaller ones and even lower than kidney donor patients, once the amount of lost functioning nephrons at surgery increases respectively. So eGFR changes after radical and partial nephrectomy depends on the quality and extension of the remaining normal tissue, mainly in the affected kidney, and the biggest the tumor the smallest the remaining functional tissue to be preserved in NSS, making the tumor size inversely proportional to the opportunity to preserve nephrons.

In addition to a trend to lower opportunity to preserve nephrons in bigger tumors, as NSS advances towards larger specimens, the procedure itself becomes much more complex and in turn may not result in better final eGFR in some cases, although pooling studies in a meta-analysis (8) shows similar complication rates, blood loss and operative time.

In the last issue of IBJU, Alvim et al. (12) attempt to evaluate one of many aspects of how to define the limit between partial and radical nephrectomy (13). The author mentions the retrospective non-randomized aspect of the study and when researching the subject one may find that this is seen more often than not. As expected, the radical nephrectomy group had much larger specimens, usually a more aggressive pathological histology and Fuhrman grades and that will make it far more likely for the partial nephrectomy group to achieve good oncological outcomes and survival rates as seen in the respective hazard ratios even after adjustment for characteristics (12). Conversely, it is not prudent to argue with a surgeon that decides to convert a partial nephrectomy into a radical one when it is a safer oncologic choice or technically overwhelming, but that shifts the patient characteristics towards having to compare tumors of different sizes, with greater aggressiveness to smaller and more indolent ones.

The limits for partial nephrectomy depend on many complex aspects involving not only the tumor but also the patient characteristics. The EORTC 30904 randomized trial (14) showed that the simple comparison between partial and radical nephrectomy would not answer most of our questions. Large randomized, prospective studies with follow up that comprise not only oncologic results but are also long enough to perceive the renal function potential advantage of NSS in complex and advanced tumors, and also studies matching for renal damaging features like hypertension, obesity and diabetes, are still missing.
